# Obesity Animal Models for Acupuncture and Related Therapy Research Studies

**DOI:** 10.1155/2021/6663397

**Published:** 2021-09-30

**Authors:** Xuwen Zhang, David Val-Laillet

**Affiliations:** ^1^Guangzhou University of Chinese Medicine, Guangzhou, China; ^2^Panyu Central Hospital, Guangzhou, China; ^3^INRAE, INSERM, Univ Rennes, Nutrition Metabolisms and Cancer, NuMeCan, Rennes, St Gilles, France

## Abstract

Obesity and related diseases are considered as pandemic representing a worldwide threat for health. Animal models are critical to validate the effects and understand the mechanisms related to classical or innovative preventive and therapeutic strategies. It is, therefore, important to identify the best animal models for translational research, using different evaluation criteria such as the face, construct, and predictive validity. Because the pharmacological treatments and surgical interventions currently used for treating obesity often present many undesirable side effects, relatively high relapse probabilities, acupuncture, electroacupuncture (EA), and related therapies have gained more popularity and attention. Many kinds of experimental animal models have been used for obesity research studies, but in the context of acupuncture, most of the studies were performed in rodent obesity models. Though, are these obesity rodent models really the best for acupuncture or related therapies research studies? In this study, we review different obesity animal models that have been used over the past 10 years for acupuncture and EA research studies. We present their respective advantages, disadvantages, and specific constraints. With the development of research on acupuncture and EA and the increasing interest regarding these approaches, proper animal models are critical for preclinical studies aiming at developing future clinical trials in the human. The aim of the present study is to provide researchers with information and guidance related to the preclinical models that are currently available to investigate the outcomes of acupuncture and related therapies.

## 1. Introduction

Obesity, which is now considered as a pandemic, is highly prevalent in America and European countries. According to the 2016 data from the WHO, 36.2% of adults in America were obese, 22.3% in Germany, and 21.6% in France [1], whereas 18% of children and adolescents worldwide were considered as overweight or obese. Obesity, which is basically defined as a pathological increase in bodyweight and fat mass, is also related to many diseases such as type-2 diabetes, cardiovascular diseases [2], subfertility [3], bone microarchitecture [4], and even to some forms of cancer [5]. Obesity is also frequently associated with eating disorders and psychological problems that complicate the clinical picture and increase the risk for further health problems. The cost and energy to treat these diseases represent a huge financial and societal burden. There is still no consensus treatment recognized as effective and completely safe. Acupuncture and electroacupuncture (EA) are gaining more and more attention for their health outcomes and little adverse effects, though research exploring the outcomes of these therapies cannot be performed exclusively in humans because of ethics and practical constraints and because mechanistic studies are required, with the necessity to sample biological tissues when necessary. Some factors such as diet and physical exercise are also much easier to control in animal models compared to human volunteers.

Obesity is a multifactorial and complex disease involving metabolic disorders, neurohormonal-altered processes affecting many organs such as the gut, liver, and brain, as well as pathological behavioral features, and quite often a low-grade chronic inflammation. There are many kinds of animal models that have been used for obesity research, including rodent models such as mice and rats [6], as well as large animals such as the pig, dog, sheep, macaque, and other nonhuman primate species [7] and even nonmammalian models such as the zebrafish [8] and drosophila [9]. The ways to induce obesity models (referring to their construction validity) are also various, such as high-fat and/or high-sugar diet exposure, spontaneous mutation exploitation or genetic engineering, and iatrogenic induction [10]. The most frequently used obesity animal models for acupuncture and EA research studies still are the rodent models. They are very interesting models to investigate specific physiological or metabolic features of obesity and the way they can be affected by potential treatments. Because rodent gut-brain anatomy and functioning are very different from those in humans and because the analogy with behavioral and cognitive features of obesity in humans is better achieved with closer species in terms of ontogeny, large animal models should appear more relevant to study these particular questions. When it comes to localizing acupuncture points, it is obvious that analogy with humans is better achieved in large animal species that are morphologically and/or ontogenetically closer to the human.

The localization of acupuncture points is associated with specific anatomical and physiological features. Many acupuncture points are in the vicinity of major nerves, blood, or lymphatic vessels. These locations are richly innervated and have autonomic nervous associations. Such locations include nerve penetration of fascia, exits through bony foramina, neurovascular bundles, and sites of nerve branching [11]. Additionally, many acupuncture point locations are also associated with regions that generate muscular dysfunction and pain, such as myofascial trigger points or musculotendinous junctions and muscle motor points. For acupuncture and EA, it is critical to use obese animals that have relatively suitable body volumes to enable researchers to distinguish different muscles, bones, and specific anatomic locations. In this study, we will use the Chinese names to identify the acupoints of interest and will indicate the World Health Organization (WHO) corresponding codes in brackets. For animals, specific names and codes are used according to Yu [12], and we will indicate them in addition to their human equivalents if necessary.

Some points are located on the basis of anatomical landmarks, some of which presenting similarities or analogies with human points. Other points are pain points (also known as trigger points or Ashi points). In contrast to acupuncture points, pain points have no defined position. They usually occur near lesions but sometimes arise quite far away from them. Most clinicians locate the pain points by systematic pressure palpation of the spine and the paraspinal and limb musculature or stimulate the skin of the affected area and surroundings to locate pain points using a medical reflex hammer [13]. Ashi points are also located on pain or tension areas and are revealed by the avoidance reactions elicited by palpation [14]. Different from humans, animals cannot tell veterinarians where they feel pain, which requires alternative strategies to locate Ashi points. Reflex hammer or Von Frey hair, for example, can be used to investigate the animals' response to stimulation in the vicinity of painful areas, but patience and experience are needed from the veterinarians to interpret these behavioral responses.

The aim of this review is to provide some comparative data for the use of acupuncture, electroacupuncture, and related therapies in the human and different animal models including rodents, nonhuman primates, and large animal models such as pigs, in the context of obesity and related diseases. We will present the respective advantages, disadvantages, and specific constraints of these models compared to humans in relation to their face, construct, and predictive validity. Finally, research perspectives will be proposed with the aim to identify which scientific questions should be investigated in the human or in animal models, respectively, to understand how acupuncture and related therapies can be applied in clinics and which underlying mechanisms are involved.

## 2. Human Studies on Obesity and Acupuncture/Electroacupuncture

Obesity derives from a wide range of physiopathological factors in the scope of biological, socioeconomic, and cultural influences. These factors can include numerous polymorphic gene products, psychosocial, and behavioral factors, food choice, gut microbiome, and chronobiology. Obesity can be the result of a pathological process or pharmacological treatment, but it is also a risk factor for the development of many comorbid conditions [15], such as hypertension and cardiovascular morbidity. It is often accompanied by many alterations at the hormonal, inflammatory, and endothelial levels, also including anomalies of the sympathetic nervous system, renal function, and at the microvascular level. Insulin resistance is a factor that can stimulate and potentiate other pathological mechanisms [16].

Food intake control and consequently bodyweight management depend on homeostatic regulations and on complex behavioral and neurocognitive processes that take place in specific brain regions involved, respectively, in the hedonic dimension of food intake and its cognitive control. Modulating the activity of these brain structures represents a promising strategy to improve food intake control [17]. Latest approaches for obesity treatment focus on medicine targets in the central nervous system (e.g., the leptin-melanocortin axis, the opioid system, GLP-1/GLP-1 system, and FGF21/FGFR1c/b-Klotho axis), but only a few investigational agents will be able to meet the FDA regulatory criteria and advance onto the marketplace [18]. Other strategies are explored, such as nonpharmacological approaches (e.g., microbiome replacement or supplementation), but all these strategies have modest effects, and their long-term safety and adverse effects need to be further investigated [19]. The exploration of safe therapies, as an alternative or in combination with other treatment strategies, is consequently needed.

Acupuncture, electroacupuncture (EA), and related therapies have been widely used to treat obesity in clinics (Table 1). Related research studies have reported positive outcomes, with or without concomitant interventions in terms of diet and physical activity. Based on effective evidence of acupuncture treatment on human drug/alcohol addiction [54–56], Chen et al. [57] proposed a potential treatment role for food addiction and obesity since drug addiction presents evident similarities with food addiction [58]. Although, there is no human or animal study addressing specifically the effectiveness of acupuncture in treating food addiction, many studies have supported the use of acupuncture or EA for obesity treatment. The mechanisms are still unclear, but neuroendocrine regulation may play an important role [59].

Previous research studies reported different cerebral response patterns following the stimulation of acupuncture points in different spinal segments. Zusanli/Sanyinjiao (ST36/SP6) stimulation in healthy human subjects activated the orbitofrontal cortex and deactivated the hippocampus, while Yinlingquan/Chengshan (GB34/BL57) activated the dorsal thalamus and inhibited the primary motor area and premotor cortex [60]. In healthy right-handed women, acupuncture at Zusanli (ST36) increased the anterior insula hemodynamic signal and decreased that in limbic and paralimbic structures including the amygdala, anterior hippocampus, and the subgenual and retrosplenial cingulate cortices, while only EA activated the anterior middle cingulate cortex [61]. In healthy right-handed adults, acupuncture at Hegu (LI4) and Zusanli (ST36) increased brain activity in the hypothalamus and nucleus accumbens, while deactivating the rostral part of anterior cingulate cortex, amygdala formation, and hippocampal complex, in comparison to a sham procedure consisting in superficial pricking on nonacupuncture points on the leg [62]. We can notice that Zusanli (ST36) is a classic point in acupuncture and EA but the outcomes depend on the type of patients and protocols.

Research studies were also performed in subjects with bodyweight management problems. In overweight humans, acupuncture on Yinlingquan (SP9) and Zusanli (ST36) decreased the hypothalamus-thalamus functional connectivity, which was negatively correlated with hunger. It was suggested that the increased dopamine modulation during acupuncture was possibly associated with the decreased poststimulation limbic system and spinothalamic tract connectivity. This correlation was absent under sham stimulation with a similar procedure where needles were removed immediately [53]. More recently, Ren et al. [52] investigated in overweight/obese adults the acute and long-term (6–8 weeks) brain responses to acute EA and sham stimulation (superficially to a depth less than 5 mm and 2 cm adjacent to the selected acupoints). They found significant differences between groups in terms of resting-state brain activity and functional connectivity, in the acute and long-term conditions, and in brain areas involved in the inhibitory, hedonic, and/or cognitive control of food intake, gastric motility, and satiety control (e.g., dorsolateral and ventrolateral prefrontal cortex, orbitofrontal cortex, insula, and precuneus). EA treatment was also associated with significant weight loss. Although these results are very interesting, it is important to specify that 16 acupuncture points (distributed on the abdomen, legs, and arms) were used in combination, which makes impossible to discuss their respective roles and importance for obesity treatment. Despite the clinical value of these data, they are not sufficient to describe and support the mechanisms underlying the observed outcomes. Very few studies demonstrated the effects of acupuncture and related therapies on the nervous system and especially brain functioning. Clinical trials are facing strict ethics and recruitment constraints, which makes difficult the comparison between different strategies in terms of acupoints combination or stimulation mode. This is why preclinical studies in relevant animal models are needed.

## 3. Rodent Models of Obesity and Acupuncture/Electroacupuncture

Rodent models present many anatomical and physiological differences compared with humans; however, we cannot ignore their wide use in obesity research and remarkable inputs to biomedical research in general. First, there are many kinds of obesity rodent models, including genetically engineered models and diet-induced models based on the consumption of high-energy diets that are very often high-fat diets. Second, rodent models are cheaper and easier to use for animal experimentation than large animals and nonhuman primates. Regulations and ethical rules framing rodent experimentation are also less restrictive that those framing the use of nonhuman primates. Eventhough rats and mice are rather close in terms of phylogeny, they present many differences (e.g., technical considerations, basic functional differences, social, addictive, and impulsive behaviors) leading to potentially different experimental outcomes [63], which need to be considered in the context of acupuncture and related therapies research.

From a “construction validity” point of view, diet-induced obesity is the most natural way to mimic modern obesity epidemic in developed countries, which mainly takes its roots in the chronic consumption of high-energy palatable foods. Swiss mice [64], C57BL/6NCrl mice [65], and C57Bla6J mice [66] can easily declare obesity after about 10–19 weeks of high-calorie diet. Sprague–Dawley rats fed with a high-fructose diet during 8 weeks showed some metabolic disturbances similar to what is described in human obesity, e.g., ectopic lipid deposition, altered hepatic insulin sensitivity, and increased de novo lipogenesis [67]. In Wistar rats, the metabolic effects of a high-energy diet are more pronounced and detected earlier than in Sprague–Dawley rats. These effects include increased weight gain, body fat mass, mesenteric adipocytes' size, adiponectin, and leptin plasma levels, as well as decreased oral glucose tolerance [68]. The gut microbiota of these obese rats also presented more abundant *Bacteroides* and *Prevotella* taxa, but less *Bifidobacterium* and *Lactobacillus* compared to SD rats, which may represent an important vector of metabolic disturbances [68], considering the role of the microbiota-gut-brain axis in the emergence of metabolic diseases [69]. The offspring born from pregnant SD rats, 50% food restricted from gestational day 10 to term, initially presented abnormal lipogenesis before declaring obesity during the period of catch-up growth [70]. The adipose tissue is the first site to exhibit increased de novo synthesis and desaturase activity [70].

Obesity rodent models can also be achieved by specific genetic crossing. Spontaneously hypertensive stroke-prone SHRSP/IDmcr-fas rat is established by crossing Izumo SHRSP rats (SHRSP/Izm) with Zucker fatty (fa/fa) rats. These rats exhibit multiple obesity risk factors, including severe hypertension, obesity, and hyperlipidemia [71]. Otsuka Long-Evans Tokushima Fatty (OLETF) rat is an early onset overeating-induced obesity for spontaneous mutation, characterized by the lack of expression of functional CCK1 [72]. Zucker rats are hyperphagic, especially towards high-fat food [73], and have lower levels of locomotor activity [73], which mimics the sedentary habits usually observed in human obese subjects. They have mutated leptin receptor genes [74], i.e., two homozygous fa/fa “fatty” alleles resulting in leptin insensitivity, which is related to hyperphagic phenotype and higher appetite [75]. KK/HlJ (KK inbred strain was inbred by Kuo Kondo in 1944. The KK/HlJ substrain was provided by Dr. Leiselotte Herberg, Diabetes Research Institute, Düsseldorf, Germany) mice are a polygenic obese mouse model with elevated serum iron levels. Since adipose tissue remodeling is concomitant with high iron levels, this causes local adipose tissue insulin resistance [76]. db/db mice are an obesity model of lost function due to mutation in the gene encoding leptin receptor. It is characterized by obesity, insulin resistance, severe hyperglycemia, pancreatic injury, and cardiovascular complications, which support a model of metabolic disorder syndrome induced by obesity [77]. db/db mice present hippocampal neuroinflammation, which is consistent with obese chronic inflammation [78]. Stat5NKO mice are characterized by Stat5 locus deletion in the CNS, which triggers the development of severe hyperphagia-induced obesity, with impaired thermal regulation in response to cold, hyperleptinemia, and insulin resistance [79]. TRPV1 knockout (TRPV1−/−) mice exposed to high-fat food present more serious glucose intolerance, cardiac oxidative stress than normal mice [80].

Over ten years of acupuncture and EA research studies, obesity rodent models were the most widely used animal models. According to previous research studies, acupuncture in rodents showed positive outcomes, though these outcomes often differ from what has been described in humans. Among the rodent models used for acupuncture and EA studies, Sprague–Dawley rats and Wistar rats are the most represented (Table 2). Both strains have similar body size and acupoints localizations [89, 97, 113]. EA at Zusanli (ST36) in SD rats reduced bodyweight and inflammatory responses such as TNF-*α*, IL-6, and IL-1 levels in serum as well as mRNA expression in adipose tissue [90]. In the genetic Zucker rat obesity model, EA treatment at Zhongwan (RN12) and Guanyuan (RN4) acupoints led to significantly lower serum leptin and higher adiponectin/leptin ratio compared to an obese control group [114]. The decreased leptin may improve insulin sensitivity and increase insulin-stimulated glucose uptake in adipocytes [115]. Acupuncture at Zhongwan (CV12), Tianshu (ST25), Qihai (CV6), Ganshu (BL18), (Pishu) BL20, and Shenshu (BL23) significantly reduced blood glucose levels, with no effect on bodyweight [105]. EA at bilateral Zusanli (ST36) and Neiting (ST44) in Stat5NKO obesity mice reduced bodyweight, decreased plasma concentration of glucose, and reversed the altered gene expressions in the hypothalamus and epididymal white adipose tissue [109].

For some obesity rodent models, we found no example of acupuncture studies: this is the case for SHRSP/IDmcr-fas rats [71], db/db mice (loss of function mutation in the gene encoding leptin receptor) [78, 116], KK/HlJ mice [76], high-fat diet induced obesity Swiss mice [64], and C57BL/6NCrl mice [65].

Traditional Chinese veterinary texts provide information on acupuncture points only for large animals. There is still no systematic acupuncture points' description in rodents. The situation differs in large animals such as pigs, sheep, horses, or dogs, for which veterinarians accumulated experience in using acupuncture or EA treatment. There are no veterinary medicine acupuncture textbooks describing acupoints on rats or mice. Most acupuncture or EA research studies using rodent models benefited from previously published studies for the acupoints localization [85, 110] or adapted the definition of rodent acupoints on the basis of the human anatomical acupoints location [88, 104]. To date, we found no clear and systematic description of rodent acupoints in the general and scientific literature [96, 112], besides the fact that many acupoints cannot be identified in rodents because of significant anatomical differences compared to humans. Humans sleep and rest at night, which is consistent with the meridian work (circular motion) time dependent on the circadian rhythm, but rats and mice, as nocturnal species, normally exercised at night and sleep during daytime. In rodents, the small body size and thin muscle layer protecting the internal organs complicate acupuncture or EA because the risk for hurting pleural cavity or nerves in the leg is increased, which might lead to muscle atrophy. Unlike large animals, such as pigs [117], there is still no research about the description of rodent meridians, which makes more difficult and uncertain the implementation of acupuncture in these models. As a consequence, it is more difficult to interpret the outcomes of acupuncture or EA in rodents and to discuss the interindividual variability in terms of success or failure.

Rodents have a small and lissencephalic brain that makes brain explorations more difficult, technically speaking, and less relevant in terms of analogy with human. It is also more difficult to distinguish different muscles and nerves in rodents and to identify the precise spinal segments location for acupuncture points, especially in mice. This method is used for precise stimulation or inhibition of neuronal pathways in freely moving animals and is based on the transfection of a specific set of neurons with specific light sensitive proteins that can subsequently be activated by illumination [118, 119]. In rodents, it is easier to perform repeated behavioral tests with many subjects than in large animals and nonhuman primates [120]. In the context of operant conditioning (lever pressing) with delayed rewards as executive function, obese Zucker rats learned to press the liver more quickly than lean animals. Their progressive ratio breakpoints (a measure of reward efficacy) were as high as that of the lean group [121]. But in contrast with human, obese individuals were more likely to be present-focused, choosing smaller immediate rewards rather than larger delayed rewards (excessive delay discounting) [122, 123].

## 4. Nonhuman Primate Models of Obesity and Acupuncture/Electroacupuncture

Primates are the animals closest to humans in terms of phylogeny, metabolic physiology in the major sites of lipogenesis (adipose tissue and liver), and lipoprotein subclasses circulation, physiology of thermogenesis, and insulin-meditated glucose utilization [124]. Eight weeks of high-fat high-sugar diet is enough to increase fat mass and plasma triglycerides and reduce circulating adiponectin concentrations in baboons (*Papio hamadryas*) [125]. Feeding rhesus monkeys with fructose-sweetened solution daily during 6–12 months also induced many metabolic perturbations including central obesity and type-2 diabetes [126]. High-fat maternal diet during pregnancy induced a dysbiosis in macaque's offspring, and these persistent alterations occurred despite cohousing of juvenile cohorts [127], which is coherent with results obtained in mice, where diet-induced maternal obesity can affect the progeny's gut microbiota, with persistent effects until young adulthood [128]. Like human without any diet restriction, free-ranging rhesus monkeys can also spontaneously develop obesity and diabetes on the island of Cayo Santiago [129, 130]. Female cynomolgus macaques have been observed to develop spontaneous obesity in adulthood [131], with bodyweight showing positive correlation with increased serum leptin levels [132]. This trend is consistent with the correlation observed between the ob gene (which encodes leptin) expression and the percentage of body fat in humans [133]. With constant diet and environment, only part of the population progresses to diabetes, thus implicating genetic susceptibility factors that have not yet been described. Three novel single nucleotide polymorphisms (SNPs), two in apolipoprotein B (APOB) and one in phospholipase A2 (PLA2G4A), have been found associated with persistent weight stability and insulin sensitivity in lean macaques, which collectively produces an obesity-resistant phenotype in adult female macaques [134]. A recent human meta-analysis of body mass index (BMI) genome-wide association studies estimated that 97 loci accounted for approximately 2.7% of BMI variation, and common variants accounted for up to 21% of BMI variation [135]. However, potentiating factors in human (i.e., diet, exercise, early life exposures, reproductive life-stage, sex, and comorbid metabolic conditions) make difficult to clearly define obese-susceptible and obese-resistant genomic variants.

Important similarities in the frontal cortex organization have been found in humans and other primates, even in the case of regions assumed to support human-specific functions. Areas in the human medial frontal cortex, including areas associated with high-level social cognitive processes such as theory of mind, showed a surprisingly high degree of similarity in their functional coupling patterns with the frontal pole, medial prefrontal, and dorsal prefrontal convexity in the macaque [136]. In capuchins and macaques, high-degree expansion was found in the temporal parietal junction, ventrolateral prefrontal cortex, and dorsal anterior cingulate cortex, all of which being involved in complex cognitive and behavioral functions. These expanded maps correlated well with previously published macaque-to-human registrations [137]. Between human and nonhuman primates, gray matter presents no significant difference, in relative terms, but the prefrontal white matter shows the largest differences and may have played an important role in human brain evolution [138]. From 358 dense individualized and common connectivity-based cortical landmarks (DICCCOL), 65 DICCCOLs are common in macaque monkey, chimpanzee, and human brains and demonstrated the consistencies of anatomical locations and structural fiber connection patterns between these species [139–141]. Furthermore, the monkey brain is overall structurally as variable as the human brain; low variability areas may have evolved less recently and have more stability, while high variability areas may have evolved more recently and be less similar across individuals [142].

Nonhuman primate models have been used in acupuncture research and provided interesting outcomes. The strong anatomical analogy with humans facilitates the localization of acupoints, which makes possible to identify more corresponding acupoints than in rodents, for example. In primate acupuncture research studies, the acupoints locations were referenced to acupuncture points anatomy locations in the human [143, 144]. Chimpanzees were successfully trained to participate voluntarily in the acupuncture treatment, which avoids the use of anesthetics during acupuncture treatment and prevents any side effects of the anesthesia [143]. This also makes acupuncture more meaningful. Previous research using acupuncture treatment on Liangqiu (ST34), Dubi (ST35), and Zusanli (ST36) showed positive outcomes in chimpanzees with osteoarthritis and notably in two individuals with severe osteoarthritis of which the mobility was significantly improved [143]. Rhesus monkeys (*Macaca mulatta*) with Parkinsonian-like symptoms also received EA on Hegu (LI4) and Zusanli (ST36) after training without anesthesia and showed improved movement speed and muscle activity between arms after EA treatment [144]. According to high possibilities in terms of training of primates, complex behavioral and cognitive tests are possible and easier to perform [145]. Though, to date, we found no acupuncture or EA experiment reported on obese nonhuman primate models.

Amongst the main disadvantages of nonhuman primate models are their very long lifespan and related housing constraints, the very strict ethical regulations framing their use for research, as well as their susceptibility to transmit zoonotic diseases. Nonhuman primates are also very expensive due to specialized housing needs, the necessity for trained and knowledgeable husbandry and technical research staff, as well as the need for specific veterinary care.

## 5. Large Animal Models of Obesity and Acupuncture/Electroacupuncture

Large animal models including pigs and sheep have been often used for obesity and nutrition research studies because they have more similar anatomical and physiological characteristics with humans compared to rodents. For example, 16-week high-fat diet exposure can make Dorset Horn greyface crossbreed sheep obese with metabolic disorders such as decreased blood-brain insulin transport, which contrasts with reports on genetically obese rats [146]. Among large animals, pigs and especially minipigs represent an economically and ethically promising substitute to nonhuman primates. Pigs have proportionally similar organ sizes and very comparable gastrointestinal tract anatomy, morphology, and physiology compared to humans and contrary to rodents [147]. Exposed to a high-fat diet, some minipig breeds easily develop weight gain and metabolic disorders, including highly specific brain anomalies comparable to those described in the human [148]. The Ossabaw swine is notably characterized with a “thrifty gene” phenotype propensity to store fat when exposed to excess calories [149]. Its increase in the transcript number and early transcriptomic alterations in the overall omental adipose tissue has been suggested to be a good model for studying childhood obesity [150].

**Figure 1:**
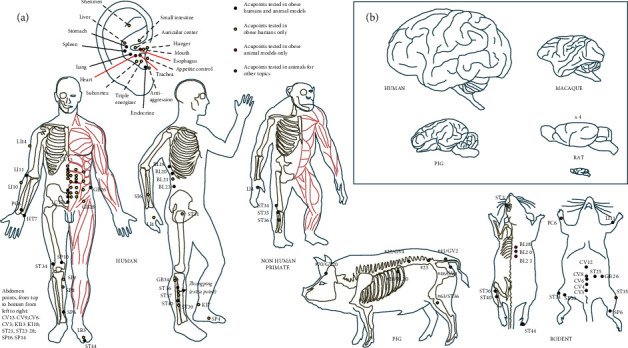
(a) Acupoints described and used in humans and different animal models including nonhuman primates, pigs, and rodents, mainly in the context of obesity and possibly in other topics. Auricular acupoints and body acupoints have been used in humans and animal models research studies. The codes for acupoints were indicated in accordance with the International Acupuncture Nomenclature proposed by the WHO. Pig acupoints were identified according to the textbook entitled “Traditional Chinese veterinary acupuncture and moxibustion” [12] and combined with human acupoints codes with comparable anatomical and structural landmarks. (b) Schematic representations of the brain in the human, macaque, pig, and rat.

Besides that, the pig brain has a convoluted or gyrencephalic cortical surface (Figure 1(b)), superficially resembling that of primates including humans [151, 152]. The pig's skull is 40% thicker and the head twice as big compared to humans, which might sometimes produce artifact susceptibility during brain imaging, but strategies can be elaborated to optimize interspecies comparisons [153]. The pig's brain remains large enough to enable the identification of cortical and subcortical structures for neurosurgery and conventional imaging techniques in living animals. Pig brain is gyrencephalic and has a folded brain cortical surface with well-defined circumvolutions, an elongated oval shape with the hemispheres being widest at the posterior third and the occipital pole being larger than the frontal pole [154]. The olfactory system is more developed than in humans and occupies a large portion of the anterior part of the brain [151]. The brain cortex, including the somatosensory (SI) and prefrontal cortex (PFC) as well as their connections, the basal ganglia, and hypothalamus have been clearly described [138, 155]. Recent anatomical research studies showed that the telencephalon of the Göttingen minipig cerebrum covers a large surface area, including neocortical gyrencephalic and ventral subrhinal parts, the first part being located dorsal to the rhinal fissure and the second part being dominated by olfactory, amygdaloid, septal, and hippocampal structures. The inner subcortical structure of the minipig telencephalon is dominated by a prominent ventricular system and large basal ganglia, wherein the putamen and the caudate nucleus posterior and dorsally are separated into two entities by the internal capsule, whereas both structures ventrally fuse into a large nucleus accumbens [156]. All these brain structures are primary regions of interest for the exploration of the brain correlates of food intake control. In comparison, the small lissencephalic brain of rodents makes more difficult functional imaging and translation to humans.

In the Göttingen minipigs, the prefrontal cortex activity was reduced after 5 months of ad libitum Western diet, which closely resembles brain anomalies previously described in obese humans [148]. Fed ad libitum during 41–47 months, Göttingen minipig also showed a deregulation of several obesity and inflammation-relevant protein-coding genes and miRNAs, of which many are also known to be deregulated in obese humans [157]. The minipig's offspring exposed to prenatal restricted nutrition [158] or maternal Western diet [159, 160] demonstrated obesity symptoms such as lower basal brain activity in the prefrontal cortex, cognitive and hedonic brain processes alterations, as well as negative changes in behavior and metabolism. All these studies demonstrate that minipig models are highly adapted to study the impact of obesity in the young or adult age, as well as the role of deleterious nutritional conditions during the perinatal period on further metabolic and behavioral imprinting.

Different pig models were created and used to investigate genetic factors related to obesity and its comorbidities. For example, a F2 pig generation was created upon a cross between lean production pigs (Yorkshire/Duroc)  ×  Göttingen minipig before being extensively phenotyped for 36 obesity-related traits, revealing large phenotypic and genetic variations [161]. There are also genetically engineered pigs, such as transgenic miniature pigs expressing human ApoCIII, which were generated by the transfection of somatic cells combined with nuclear transfer. Transgenic pigs showed significantly higher triglyceride levels and delayed clearance of plasma triglycerides, accompanied by significantly reduced lipoprotein lipase activity in postheparin plasma [162]. Lp-phospholipase A2 (PLA2) transgenic swine has higher triglyceride levels and inflammatory gene, IL-6, MCP-1, and TNF-*α* mRNA in peripheral blood mononuclear cells. It was generated by eukaryotic expression plasmid and via somatic cell nuclear transfer. The overexpression of Lp-PLA2 was driven by EF1-*α* promoter [163].

In the context of behavioral tests, pigs usually have better performances than rats, for example, during motivation tests with a progressively increasing ratio between efforts and rewards [164]. Domestic pigs have the ability to discriminate between food sites of different relative values and to remember their respective locations. They are able to adjust their behavior when exposed to food sites with different food quantity and profitability [165]. Minipigs perform very well during cognitive tests such as the spatial hole-board discrimination task, the alley maze test, or two-choice food tests. In these paradigms, maternal Western diet piglets showed behavioral differences in comparison to maternal standard diet piglets, such as higher stress and lower performance in the alley maze [159]. It is consequently a good model to assess nutrition-related behavioral outcomes. In OLETF rodents (a genetic obesity rat model characterized by spontaneous mutation, lacking expression of functional CCK1), lickometers were used to assess sugar preference, the interest in sucrose appearing only during early adulthood. The animals' preference for the higher sucrose concentrations was exacerbated along time and age [72]. The reason is still unknown, but the progressive maturation of the orosensory and hedonic systems, as well as the dopaminergic system mediating the reward value of sweet foods [166], might explain this difference in comparison to species with a quickest brain maturation, such as pigs. It has been demonstrated that juvenile pigs exposed to aversive or preferred foods present specific brain activity, especially in the reward circuit, demonstrating that they are perfectly able to discriminate the hedonic value of food [167]. Metabolic differences were notably observed in neural circuits known to be involved in humans in the characterization of food palatability, eating motivation, reward expectation, and more generally in the regulation of food intake [152]. In humans, exposure to flavors with different hedonic values induced eating motivation, reward expectation, and food intake regulation differences, all these processes being controlled by several brain structures including the amygdala and insular cortex [168]. Piglets also have higher acceptance of flavors that were present in the sow's diet during gestation and lactation [169], which is consistent with what has been described in human babies [170].

The precise description of acupoints in pigs, equine, bovine, porcine, sheep, camel, canine, and rabbit can be found in the textbook entitled traditional Chinese veterinary acupuncture and moxibustion [12], which has accumulated many years of veterinarians' clinical experience. Large animals such as pigs, cows, and dogs have good reactions to acupuncture, with positive health outcomes. In veterinary medicine, acupuncture has been widely used to treat swine diseases [171]. For instance, Changqiang (GV1), Yaoshu (GV2), Mingmen (GV4), and Baihui (GV20) are the usual choice to treat impotence and penile paralysis in male boars [172]. In the early stage of inoculation with *Escherichia coli*, acupuncture treatment on Changqiang (GV1), Baihui (GV20), Pishu (BL20), and Zusanli (ST36) had a better effect than neomycin on diarrhea and gut inflammation in young pigs [173]. A two-week treatment on Dafengmen (#70), of which the anatomical location is similar to human Baihui (GV20), significantly improved sleep quality in pigs and changed the catecholamine levels in pooled urine [174]. Tianzhu (BL10) and Dazhu (BL11), as well as Fengchi (GB20) and Jianjing (GB21) have a good effect on paralytic or lame dogs [172]. Hemoacupuncture (a technique encouraging a small amount of bleeding from specific acupuncture points) at Shaoze (SI1), Guanchong (SJ1), Shangyang (LI1), and Qiantitou, as well as acupuncture on Shaoshang (LU11), Shaochong (HT9), Zhongchong (PC9), and Qiantimen, had positive effects on horse with chronic laminitis [175]. Twelve weeks of acupuncture at Baihui (GV20) and Ashi points in mildly lame horse improved horses' gaits to an appreciable degree by objective and subjective analyses [14]. The injection of 125 *μ*g/m^2^ dexmedetomidine in acupuncture point Baihui (GV20) in dogs increased the duration and degree of sedation and analgesic effects of dexmedetomidine compared with intramuscular injected in loci that did not correspond to acupuncture points [176]. After 24 weeks of treatment, acupuncture alone and combined with analgesics both improved Helsinki chronic pain index (HCPI), visual analog scales (VAS), and improved life quality in 181 dogs with neurological and musculoskeletal diseases [177].

Because the blocking of low hydraulic resistance channel of the minipig stomach meridian by gel triggered gastric and intestinal distension, it has been suggested that minipigs have a meridian reactivity similar to humans. Indeed, according to basic Chinese medicine theory, human stomach meridian controls digestion and intestinal stomach movement [117]. In order to find in animals the best analogy with human acupuncture points, it is important to rely on anatomical landmarks and not only on the names given to the acupoints in the animal. For example, the human Baihui (GV20) acupoint is located on the midline of the head vertex. In pig, there is a so-called “Baihui” acupoint, but it is located at the top of the back near the hip and has nothing to do with human Baihui. The pig acupoint #70 Dafengmen has an anatomical location similar to human Baihui, and as in humans, its stimulation can modulate urine catecholamine and improve sleep condition [174].

Surprisingly, we found no study aimed at investigating the effects of acupuncture or EA in a pig model of obesity or hyperphagia (an abnormally strong sensation of hunger or desire to eat often leading to or accompanied by overeating) nor in any other large animal model, which highlights the complete lack or research in this promising field. We recently published a hypothesis study [178] presenting the rationale and a methodological approach to investigate the effects of electroacupuncture in obese minipigs. This ongoing study is the first of its kind. The acupuncture points that were selected for this work included three combinations of paired acupoints, i.e., Pishu (#28) and Liumai (#27) on the back, Dafengmen (#70) on the head, and Sanwan (#35) on the abdomen, as well as Hangou (#79) and Housanli (#63) on the hind legs. Our preliminary results obtained in normal weight animals showed that acute EA did not modify the glucose and insulin plasma levels, but had a tendency to modulate the heart rate variability (HRV) assessed via electrocardiography (ECG), which is a proxy for vagus tone. Most interesting is the fact that the combination Dafengmen/Sanwan produced significant BOLD fMRI activation of the prefrontal cortex, striatum, hippocampus, and cingulate cortex, in comparison to the other combinations or a sham treatment (Zhang and Val-Laillet, in preparation). This combination is currently being tested in obese Yucatan minipigs in the context of a one-month chronic treatment with 3 EA sessions per week.

## 6. General Discussion and Conclusions

### 6.1. About the Heterogeneity of Human Clinical Trials: A Rational for Preclinical Studies

Acupuncture and EA are being increasingly popular in clinical and preclinical research. Because of the ethical, financial, and practical constraints associated with randomized controlled trials (RCT), not all research on acupuncture and related therapies can be performed in the human. Preclinical explorations are needed before the onset of RCT, and the use of relevant animal models is consequently justified. Animal studies also have the advantage to better control factors that are extremely variable and difficult to assess in human populations. Human subjects usually present diverse degrees of cooperation or understanding during a clinical trial, and it is often difficult to survey precisely their food intake and physical activity. A recent review highlighted the relative ambiguities regarding the role of physical activity in obesity treatment, and its impact on body composition sometimes shows some inconsistencies [179, 180]. Many clinical studies fail to adequately measure or assess calorie intake, concurrent changes in habitual activity, non-prescribed weight-loss behavioral strategies, or inconsistent dose (duration and intensity) of physical exercise across intervention types. Incomplete data collection further contributes to the methodological heterogeneity between clinical trials. The question of sham and real acupuncture still has not reached consistent conclusions either. Some authors show that sham stimulations (noninsertive acupuncture or nonacupuncture points) do not differ from real acupuncture [181], while others, for example, demonstrated in obese women some positive effects of acupuncture and ear point seeded compared to sham treatment on fasting insulin, HOMA-IR, and inflammation factors [27]. Further research is needed on this topic, and the question of sham acupuncture is probably much easier to treat in animal models than in humans who are subjected to psychosomatic factors and other cognitive biases towards the outcomes of a medical treatment. As highlighted in Table 1, various types of sham treatments have been used in the human, which complicates the comparison between studies.

Similarly, the multiplicity of disease models and combinations of acupoints selected makes very difficult a systematic comparison of the outcomes, which can significantly differ between studies and species. There is still no consensus guidance to select acupuncture points for obesity treatment, and many questions about the underlying physiological and behavioral mechanisms of these effects remain unanswered. From previous research studies, different acupuncture points can produce different effects. As obesity derives from complex metabolic and neurobehavioral processes including many parameters and potentially generating diverse pathological profiles, further research is needed to describe precise candidate acupoint targets and their related effects in terms of physiology and behavior. To investigate pathological and gene mechanisms, tissue sampling is often mandatory, which is difficult to perform in humans but very easy in animal models. Several authors highlighted the important role of the central nervous system and the communication pathways though it is possible to leverage brain processes in the effects of acupuncture or EA stimulation on diseases [182]. Such research usually requires histological and molecular biology studies to understand the complex mechanisms underlying these outcomes.

### 6.2. Interspecies Comparison about Ear Acupuncture and Related Therapies

From acupuncture research in humans (Table 1), we found that ear acupuncture points (Figure 1(a)) have been widely investigated in clinical trials, with positive outcomes in obese humans in terms of appetite, bodyweight and BMI, plasma lipidemia, as well as ghrelin and leptin levels, for example [22, 31, 34, 39, 47]. The reasons for these outcomes are still unknown but are probably related to complex neurophysiological mechanisms. In the human, the cymba conchae stimulation in the external ear by continuous wave significantly activates the classical central vagal projections (widespread activity in the ipsilateral NTS, bilateral spinal trigeminal nucleus, dorsal raphe, locus coeruleus, and contralateral parabrachial area, amygdala, and nucleus accumbens) [183]. All these brain regions are involved in homeostatic and hedonic food intake control. In rats, stimulation at auricular points pylorus (CO3), lung (CO14), trachea (CO16), stomach (CO4), esophagus (CO2), endocrine (CO18), and heart (CO15) has been reported to activate the hypothalamic ventromedial nucleus satiety center and reduce bodyweight [99]. Other research studies related to auricular acupuncture in obesity animal models are very scarce because rats and mice have very small ears. Mice are characterized by “microtype” ears with microtype *mallei* associated with high-frequency hearing which is not found in humans [184]. Though, some authors demonstrated in obese rats, for example, that auricular acupuncture stimulation reduced bodyweight [99], modulated feeding-related hypothalamic neuronal activity [98], reduced epididymal WAT, and increased brown adipose tissue (BAT) weight, serum norepinephrine, mRNA expressions of *β*3-adrenoceptors, and UCP1 of the BAT [185]. Compared to rodents, pigs have large ears, of which the general anatomy is more likely comparable to those in humans. Also, the obtained transfer function is congruent to human [186]. Though, we found only one study using ear acupoints in combination with other body acupoints in this species, with beneficial effects in controlling *Escherichia coli* diarrhea [173]. Most nonanthropoids primates have tall and narrow ears, the ears of monkeys and apes being more equal in terms of height and width [187].

### 6.3. Bridging the Gap between Classical Animal Models and Humans in EA Research

As highlighted in Table 2, we found that acupuncture or EA research studies in rodents were limited to several acupuncture points, especially Zusanli (ST36), because the significant differences with humans in terms of body surface/volume and anatomical structures make difficult to find more analogies with the acupuncture points in humans (Figure 1(a)). In rodent obesity models, acupuncture or EA also work well on anthropometric index and biological indicators (Table 2). But first, some rodent models, especially genetically engineered rodents, cannot explain modern human epidemic obesity with complex triggering factors (diet habits, environmental pressures, and genes), therefore, limiting their construct validity. Second, some RNA or specific protein changes after EA or acupuncture in obesity have not been confirmed in humans, and the visible changes that can directly explain acupuncture or EA effects on brain reactivity, for example, are still very rare in rodent models because of methodological and resolution restrictions, which limit their face validity. Third, some acupuncture strategies that were successful in reducing bodyweight in the human [39] did not do so in rats [104, 105], therefore, limiting the predictive validity of this model.

Genes may play an important role in the onset of obesity, and converging data were obtained in several species. The OLETF rat, with spontaneous mutation characterized by lacking the expression of functional CCK1, is an early onset overeating-induced obesity model [72]. Though, there is still no evidence of the existence of a CCK1 mutation in diet-induced obesity human, which limits the extrapolation of data from this rat model. In macaques, three novel SNPs have been found associated with obesity-resistant phenotype [134]. In humans, 22 obesity-related candidate SNP markers have been found through DNA sequence analysis by using web service SNP_TATA_Comparator and keyword articles search [99]. In rodents, in intersubspecific backcross population between C57BL/6JJcl (B6) and wild *Mus musculus castaneus* mice, Gcg and Grb14, Ly75, and Itgb6 were suggested candidate genes, but these genes had nonsynonymous SNPs [188]. Genetically engineered models can produce different results for the same parameters. For example, in genetically Zucker obese rats, blood-brain insulin transport is decreased [189], which contrasts with data in large animals. For example, decreased proportional blood-brain insulin transport was associated with weight loss in obese sheep, but the central insulin resistance (in terms of food intake response) resulted from intrahypothalamic insensitivity rather than impaired blood-brain insulin transfer [146].

Undoubtedly, nonhuman primate obesity models are closer to humans and allow for better analogy when investigating the effects of acupuncture or EA, but the higher cost and ethical restrictions limit their use. Rodent models also have advantages, since they are easily bred, housed, and handled at lower costs and with less ethical restrictions than larger animals. It is also very easy to produce genetically engineered obesity models. It should be noted also that small animal models such as mice can represent an asset for certain techniques, such as optogenetics, in order to investigate the effect of acupuncture or EA regulation on the neural basis of pathological behavior, for example [190]. Rodents and especially mice have a thinner skull compared to other large animals and are easier to handle. The recent development of optogenetics permits real-time manipulation of region and cell type-specific neural pathways in awake behaving rodents [191, 192]. Optogenetics has been used to reversibly control depression-related phenotypes in mice by manipulating the medial prefrontal cortex (mPFC) [193], for example, and further implementation of this technique in the scope of EA research should be investigated.

To date, obesity animal models for acupuncture and related therapies are restricted to rodents, which significantly limits the extrapolation and translation to human. It is, therefore, necessary to propose alternative preclinical strategies, benefitting from innovative obese animal models to support further acupuncture research studies. Large animal obesity models appear like a bridge between rodents and nonhuman primates. Pigs, especially minipigs, are closer to humans than rodents, less expensive, and difficult to breed than nonhuman primates. This species also has been extensively used for nutritional and neurosciences studies, making possible crossed obesity and brain research combined with complex behavioral explorations [147, 151, 152]. They also are very interesting for behavioral studies aimed at investigating the different dimensions of eating behavior, including individual preferences and choices, motivation, and the classical food liking and wanting usually explored in human subjects [194]. In humans, overeating usually emerges when food signals sensitivity and appetitive motivation to consume palatable foods overrule the inhibitory control of eating [195]. Comparable data have been obtained in the adult minipig model [148, 196], as well as in the context of the developmental origins of health and diseases [159, 160, 197], in terms of behavior and brain functioning.

On the specific question of brain reactivity to acupuncture or EA, research studies are rare and almost restricted to human research [52, 53]. The mechanisms and effects of different acupuncture points are still poorly described. The small lissencephalic brain in rodents (Figure 1(b)) significantly restricts neurobiological and neuroimaging research studies, and such studies in the human are costly and limited by practical and ethical constraints. Animal models with brain anatomical structures and functions more similar to those in humans are necessary, and the pig has a brain very close to that of macaques, for example, with many circumvolutions as in humans (Figure 1(b)). The fact that pig brain anomalies in the context of disordered eating and obesity are very similar to those described in the human is another potent argument to support the use of this model to investigate the neurobiological mechanisms of acupuncture and related therapies.

In the spectrum of obesity treatments, obesity surgery is usually reserved for severe obesity or moderate obesity accompanied with significant comorbidities. It is often the last recourse after failure of other treatments, but it is frequently associated with nutritional risks and potential complications. In addition or complement to obesity surgery, antiobesity medicine is one of the main treatments, but most FDA-approved antiobesity drugs have significant individual variations in response rates [198]. Long-term safety, potential adverse effects, and relative efficacy also are some of the main reasons why very few patients accept them. Most of these medicines can provide greater weight loss than do lifestyle changes alone, although few promising antiobesity medications are in the drug-development pipeline. The most promising drugs are novel molecules that are coagonists for multiple gut hormones including GLP-1, glucagon, and gastric inhibitory peptide [200]. Contrary to pharmacological treatments, acupuncture and related therapies have very few adverse effects, such as nausea, mild and transient syncope, and very rare events of septicaemia and hepatitis C infection [200]. Most of these rare events can be avoided with the use of disposable needles and good disinfection and sanitary conditions. Acupuncture alone or in combination with other therapies gains increasing attention, but the underlying mechanisms and the best acupoint combinations are still unknown. Further research studies with proper obesity animal models are needed to find the most effective treatment parameters and decipher their underlying mechanisms.

## Figures and Tables

**Table 1:** Overview of studies on human acupuncture and related therapies in the context of obesity.

Subjects	Treatment types and sham/control design	Acupuncture points and time frequency	Main results	References
Acupuncture and electroacupuncture				
RCT, randomized, patient- and assessor-blind, sham-controlled clinical trial, women aged ≥19, BMI ≥25 kg/m^2^	Real group (*N* = 48): manual acupuncture (MA) + EA vs. the control group (*N* = 43): sham MA + sham EA sham MA: nonpenetrating acupuncture at traditional acupuncture points. Sham EA: acupuncture at sites not corresponding to traditional acupuncture points with penetration to the same depth but no electrical stimulation	Body points: Hegu (LI4), Quchi (LI11), Sanyinjiao (SP6), Zusanli (ST36), Qihai (CV6), Zhongwan (CV12), Tianshu (ST25), and Shuidao (ST28). Time and frequency: twice per week for 6 weeks	MA and EA treatment did not affect anthropometric and serum metabolic parameters, but increased carnitine levels (C2, C4, C6, and L-carnitine)	Kim et al., 2020 [20]
RCT, single-blind, overweight women, aged 20–30, BMI ≥25	AA group (*N* = 30): auricular acupuncture vs. the sham group (*N* = 28): Similar plaster as the AA group without needle attached	Auricular points: Shenmen (TF4) and appetite control (TG2). Time and frequency: 20 minutes per time, once a week for totally 7 weeks	AA treatment reduced waist circumference, but weight loss and BMI were not significant in both groups: have the effect on mood improvement	Lillingston et al., 2019 [21]
RCT double-blind, obese adolescents, BMI 25–30	Auricular group (*N* = 32): auricular acupressure at obesity effective points with *Vaccaria* seeds vs. the sham group (*N* = 26): placebo auricular acupressure and noneffective points on obesity	Auricular points: auricular group, Shenmen (TF4), spleen (CO13), endocrine (CO18), stomach (CO4), and hunger (TG1); control group zone has no relationship with obesity, knee zone, hip-joint zone, lumber zone, thoracic-vertebrae zone, and tooth zone time and frequency. Both 8 weeks, one time one week, 5 minutes pressure one time before meals and whenever they felt hungry; ears were treated alternately	Auricular acupressure was better than the control group: decreased TC and LDL-C levels	Cha and Park, 2019 [22]
RCT, male, aged >60, with sarcopenic obesity	EA + AA group (*N* = 23): EA + oral essential amino acids vs. the AA group (*N* = 25): oral essential amino acids alone	Body points: Binao (LI14), Quchi (LI11), Biguan (ST31), and Liangqiu (ST34). Time and frequency: 20 minutes per time, once every 3 days for 12 weeks; essential amino acids orally, twice per day for 28 weeks	Both decreased BFP and increased ASM/H2; EA + AA is more effective and can increase muscle mass in a shorter time	Zhou et al., 2018 [23]
RCT, abdominal obese women, aged 21–53, BMI ≥23, WC >80 cm	EA group (*N* = 15): body points vs. the control group (*N* = 15): no intervention + maintained normal diet + usual exercise habits	Body points: Huaroumen (ST24), Shuidao (ST28), Huaroumen (ST24), Shuidao (ST28), Fujie (SP14), and Daheng (SP15). Time and frequency: three times a week for 3 months	EA treatment reduces BMI and WC as well as VAT volume and HFF	Lei et al., 2017 [24]
RCT, overweight and obese subjects, BMI ≥25	EA group (*N* = 79): body points + low-calorie diet vs. the control group (*N* = 82): low-calorie diet	Body points: Tianshu (ST25), Weidao (GB28), bilateral, Zhongwan (CV12), Shuifen (CV9), Guanyuan (CV4), Sanyinjiao (SP6), Quchi (LI11), Fenglong (ST40), Qihai (CV6), and Yinlingquan (SP9). Time and frequency: 20 minutes each time, two treatment sessions per week for 6 weeks + follow up 6 weeks	EA is better than low-calorie diet alone, serum prooxidant antioxidant balance, and reduces serum PAB values	Mazidi et al., 2017 [25]
RCT, obese women, BMI 30–40	Body group (*N* = 21): receive acupuncture at body acupoints vs. the auricular group (*N* = 17): receive acupuncture at auricular acupoints	Auricular points: antiaggression (nicotine addiction points), stomach (CO4); body points: Hegu (L14), Quchi (LIll), Tianshu (ST25), Zusanli (ST36), Sanyinjiao (SP6), Yinlingquan (SP9), Zhongwan (CV12), and Qihai (CV6). Time and frequency: auricular group: 6 sessions in every 15 days, manual stimulation of all four needles 15–20 minutes before meals for 30 seconds and 3 times one day; body group: two times in a week, totally 24 sessions, 12 weeks	Both groups: changes in weight, body mass index, body fat percentage, waist circumference, and hip circumference were statistically significant; auricular acupuncture is more effective in reducing bodyweight than body acupuncture	Yasemin et al., 2017 [26]
RCT, double-blind, overweight/obese T2DM adult patients, aged 20–65, BMI ≥25	EA group (N = 19): body EA + auricular points pressure + metformin vs. the sham group (*N* = 20): sham body EA + sham auricular points pressure + metformin sham design: maximally superficial needles on 0.3 cm laterally from acupoints and adhesive tape without seeds on auricular points	Body points: Zhongwan (CV12), Tianshu (ST25), Zusanli (ST36), Sanyinjiao (SP6), Shuifen (REN9), Hegu (LI4), Daheng (SP15), Shuidao (ST28), Guanyuan (REN4), Quchi (LI11), and Qihai (REN6). Auricular points: Sanjiao (CO17), hunger (TG1), stomach (CO4), Shenmen (TF4), endocrine (CO18), and spleen (CO13). Time and frequency: both 10 times, 30 minutes every time, every other day, 3 weeks; auricular points were treated 2 ears alternately	EA was better than sham EA, decreased BW, BMI, FBS, FINS, HOMA-IR, IL-6, TNF-*α*, leptin, GLP-1, resistin, ceramides, FFA, TG, and LDL-C and increased HDL-C, adiponectin, and serotonin	Firouzjaei et al., 2016 [27]
RCT, a pilot study, women aged 30–52, BMI >30	Dorsal group (*N* = 5): EA at dorsal segmental acupuncture points vs. the auricular group (*N* = 5): EA at auricular acupoints vs. the limb group (*N* = 6): EA at arms and legs acupoints	Body points: Ganshu (BL18), Pishu (BL20), Weishu (BL21), Shenshu (BL23), Shousanli (LI10), Quchi (LI11), and Zusanli (ST36). Auricular points: Zhongping (extra point); lung (CO14), and heart (CO14). Time and frequency: lasting 30 minutes, one EA session	Dorsal acupoints with best outcomes; dorsal and limb groups decreased fasting blood glucose but no change in the ear group; EA at dorsal acupoints may exert a beneficial effect on the glucose metabolism in obese women	Belivani et al., 2015 [28]
RCT, overweight and obese female adults, BMI ≥23 kg/m	MAMT group 1 (*N* = 28): BMI 23–25 receive manual acupuncture + massage therapy; MAMT group 2 (*N* = 20): BMI ≥25 receive manual acupuncture + massage therapy vs. the MAT group 1 (*N* = 28): BMI 23–25 receive only manual acupuncture therapy; MAT group 2 (*N* = 20): BMI ≥25 receive only manual acupuncture therapy	Body points: Tianshu (ST25), Liangmen (ST21), Daheng (SP15), Zusanli (ST36), Sanyinjiao (SP6), Quchi (LI11), Zhigou (SJ6), Zhongwan (RN12), and Qihai (RN6). Massage: rub the stomach meridian of Foot-Yangming, the Ren meridian, and the Dai meridian by the thenar eminence, and acupressure to manipulate the above abdominal acupoints with moderate pressure. Time and frequency: acupuncture treatment 30 minutes one time; massage 25 minutes each time, once per day for 21 days	Both MAMT and MAT could reduce bodyweight and BMI and no significant difference between the groups; the optimal periods for reductions were the first 4 days; MAT alone may present a reasonable option	He et al., 2015 [29]
RCT, overweight and obese adults, BMI 25–30 and BMI of ≥30	Acupuncture group 1 (*N* = 22): BMI 30–35 + acupuncture treatment; acupuncture group 2 (*N* = 34): BMI 35–40 + acupuncture treatment; acupuncture group 3 (*N* = 24): BMI > 40 + acupuncture treatment vs. the control group (*N* = 23): no treatment	Body points: Hegu (LI4), Quchi (LI11), Liangmen (ST21), Tianshu (ST25), Zusanli (ST36), Fenglong (ST40), Neiting (ST44), Sanyinjiao (SP6), Daheng (SP15), Neiguan (PC6), Taichong (LR3), Guanyuan (CV4), and Zhongwan (CV12). Auricular points: Shenmen (TF4), mouth (CO1), stomach (CO4), triple energizer (CO17), liver (CO12), spleen (CO13), endocrine (CO18), and hunger (TG1). Time and frequency: body acupuncture sessions twice weekly, each session was 30 minutes; weekly auricular acupuncture applied to each ear alternately; 6 months in total	Acupuncture combined with diet restriction reduced BW anthropometric measurement of adiposity, TNF-*α*, IL-6 and CRP, decreased creatinine, uric acid, and lipid profile (cholesterol and triglycerides), and fasting blood glucose, but there was no significant difference in urea, SGPT, SGOT, HDL, and LDL	Abou Ismail et al., 2015 [30]
RCT, single-blinded, adults aged 18–50, BMI ≥27	EA group (*N* = 36): both auricular electrical stimulation with electrodes + acupressure with seed-embedding vs. the sham group (*N* = 34): points not associated with weight reduction or obesity	Auricular points: EA group: Shenmen (TF4), stomach (CO4), endocrine (CO18), and hunger (TG1); sham group: ankle (AH3), elbow (SF3), shoulder (SF4), and clavicle (SF6). Time and frequency: EA once a week for 20 minutes, acupressure 1 min, 4 times per day, 10 weeks in total; ears were treated alternatively	No significant difference between 2 groups; EA group: TC, TG, and leptin decreased after intervention; sham group: TC, leptin decreased, and adiponectin increased after intervention	Yeh et al., 2015 [31]
Randomized crossover pilot study, single-blinded, overweight and obese adults, BMI 25–40	Acupuncture group (*N* = 19): nutritional counseling + acupuncture vs. the sham group (*N* = 16): nutritional counseling + sham acupuncture (sites were located close to the classical acupuncture points)	Body acupoints: Hegu (LI4), Quchi (LI11), Zusanli (ST356), Neiting (ST44), and Taichong (LR3). Auricular acupoints: hunger (TG1), stomach (CO4), and Shenmen (TF4). Time and frequency: 30 minutes each time, twice weekly for 6 weeks, with a 2-week wash-out period	A larger trial investigating the use of acupuncture for weight loss in those who have elevated eating and weight concerns is feasible	Fogarty et al., 2015 [32]
RCT, adults, age 18–65, BMI ≥25, male waist line ≥90 cm, female waist line ≥80 cm, abdominal obesity with spleen deficiency and exuberant dampness	Channel group (*N* = 33): acupuncture at hour-prescriptive points from 9 to 11 AM vs. the control group (*N* = 32): acupuncture at any time beyond 9–11 AM	Body acupoints: Fujie (SP14), Daheng (SP15), Xuehai (SP10), Yinlingquan (SP9), Diji (SP8), Sanyinjiao (SP6), and Gongsun (SP4). Time and frequency: 30 minutes, once every day for three courses of treatment with ten sessions	The total curative effect in the channel group was better than in the control group in reducing BW, BMI, waistline, obesity level, and clinical symptoms; there was no significant difference in WHR between the 2 groups	Wu et al., 2014 [33]
RCT, single-blinded, aged 18–55, abdomen fat mass obesity, man, BMI 0–40	Body EA group (*N* = 20): low-calorie diet + body EA vs. the auricular EA group (*N* = 20): low-calorie diet + auricular acupuncture (auricular pressing plasters with seeds) vs. the sham body group (*N* = 20): low-calorie diet + sham body EA: superficial inserts 0.5 cun cranially and 0.5 cun laterally from acupoints vs. the sham auricular group (*N* = 20): low-calorie diet + sham auricular acupuncture, auricular plasters without seeds	Body acupoints: Tianshu (ST25), Weidao (GB28), Zhongwan (REN12), Shuifen (REN9), Guanyuan (REN4), and Sanyinjiao (SP6). Auricular points: Shenmen (TF4), stomach (CO4), hunger (TG1), mouth (CO1), center of auricular (HX1), and triple energizer (CO17); ears were treated alternately; time and frequency: body EA 20 minutes one time; auricular plaster kept attached 3 days one time, both twice a week, 6 weeks in total	Both EA groups: WC and HC were reduced postintervention; compared with sham groups, decreased BMI, TFM, WC, and HC body in the EA group: more effective on WC; auricular EA group: more effective on HC	Darbandi et al., 2014 [34]
RCT, female overweight/obese students, aged 20–30, BMI ≥25	Auricular acupressure group (*N* = 25): auricular acupoints pressure with *Sinapsis alba* seeds vs. the control group (*N* = 24): no intervention	Auricular points: Shenmen (TF4), mouth (CO1), stomach (CO4), endocrine (CO18), and small intestine (CO6). Time and frequency: press those points 10 times at a rate of two times per second, 30 minutes before mealtime, three times daily for one month; ears were treated alternately	Auricular acupoints pressure decreased BW and BMI and increased self-efficacy	Kim et al., 2014 [35]
RCT, single blind, obese adults, aged ≥19, BMI ≥23	5-point group (*N* = 22): auricular acupuncture with indwelling needles at 5-point vs. the 1-point group (N = 21): auricular acupuncture with indwelling needles only at hunger point vs. the sham group (*N* = 15): needles fixed on surgical tape, needles removed immediately after insertion 2 mm at the 5 points selected for the 5-point group, and surgical tape remained	Auricular acupoints: 5-point group, Shenmen (TF4), spleen (CO13), stomach (CO4), hunger (TG1), and endocrine (CO18); 1-point group: hunger point; only time and frequency: one time attach last one week, once a week for 8 weeks; ears were treated alternately	Both auricular acupuncture treatment groups reduce BMI, BW, and BF mass compared to the sham group; the 5-point group reduced BMI by 6.1% and the 1-point group by 5.7%	Yeo et al., 2014 [36]
RCT, randomized placebo-controlled, pilot, double-blinded study, female obese adults, aged >18, BMI >25	Verum group (*N* = 28): auricular EA with P-Stim® device vs. the sham group (*N* = 28): P-Stim® dummy device had no power supply and had been grinded to leave only metal plates	Auricular points: hunger (TG1) and stomach (CO4). Time and frequency: four days in one week, for a period of six weeks. A follow-up visit was performed after 4 weeks; ears were treated alternately	Auricular EA better than the placebo group, with a decrease of BW and BMI	Schukro et al., 2014 [37]
RCT, overweight and obese adults, aged 18–55, BMI 25–45	Auricular group (*N* = 86): inserted the auricular pressing plaster with seed + low-calorie diet vs. the sham group (*N* = 83): inserted the auricular pressing plaster without seed on points with no relationship with obesity	Auricular acupoints: auricula group: Shenmen (TF4), stomach (CO4), hunger (TG1), mouth (CO1), center of auricular (HX1), and triple energizer (CO17); sham group: hip (AH5), spleen (CO13), nose (TG3), and esophagus (CO2). Time and frequency: pressure to the auricular points 30 minutes before eating for about 20 seconds; seed plasters were changed twice a week for a total of 6 weeks; ears were treated alternately	Auricular group was better: reduced anthropometric factors and anti-Hsp antibodies	Abdi et al., 2012 [38]
RCT, randomized, sham-controlled preliminary trial, female adults, BMI >30	Acupuncture group (*N* = 20): body points vs. the sham group (*N* = 20): acupuncture needles were not inserted but just applied under a tape at the same points	Body acupoints: Hegu (LI4), Shenmen (HT7), Zusanli (ST36), Neiting (ST44), and Sanyinjiao (SP6); time and frequency: two sessions of 20 min per week for a total 5 weeks	Decreased insulin and leptin levels, BW, and BMI; increased plasma ghrelin and CCK compared with sham	Gucel et al., 2012 [39]
RCT, overweight and obese adults, aged 18–55, BMI 25–40	Acupuncture group (*N* = 79): acupuncture + low-calorie diet vs. the sham group (*N* = 82): low-calorie diet + sham, superficial needle at nonacupoints 0.5 cm up and 0.5 cm laterally to the real acupoints	Body acupoints: Tianshu (ST25), Weidao (GB28), Zhongwan (REN12), Shuifen (REN9) Guanyuan (REN4), Sanyinjiao (SP6), Quchi (LI11), Fenglong (ST40), Qihai (REN6), and Yinlingqau (SP9). Time and frequency: 20 minutes each time, two sessions per week for a total of 6 weeks, + second period of 6 weeks only low-calorie diet and exercise for all participants	Anti-Hsp-antibodies only decreased in the acupuncture group; changes in lipid profiles were observed in the control group only; acupuncture combination with diet restriction was effective in enhancing weight loss and improving dyslipidemia	Abdi et al., 2012 [38]
RCT, young adults, aged 18-20, WC ≥80 cm in females and ≥90 cm in males	Control group (*N* = 28): Only acupressure vs. the experimental group *(N* = 27): acupressure + Japanese Magnetic Pearl on the ear acupoints	Auricular acupoints: Shenmen (TF4), mouth (CO1), stomach (CO4), small intestine (CO6), and endocrine (CO18). Time and frequency: once weekly for ten minutes for a total of eight weeks	Both groups decreased BW and WC; only the experimental group with Japanese Magnetic Pearls decreased WHR	Hsieh et al., 2011 [40]
RCT, overweight adolescents, aged 18–20, BMI ≥23	*Vaccaria* seeds group (*N* = 26): auricular acupressure with *Vaccaria* seeds vs. the JMP group (*N* = 24): auricular acupoints stimulation with Japanese magnetic tap vs. the control group (*N* = 26): nothing on tape	Auricular acupoints: Shenmen (TF4), mouth (CO1), stomach (CO4), endocrine (CO18), and small intestine (CO6). Time and frequency: ears were treated alternately; the tape was replaced every two or three days; 8-week study	Both auricular acupuncture treatments decreased BMI; *Vaccaria* seeds provided better outcomes; all three groups increased TC, TG, HDL-C, and LDL-C	Hsieh, 2010 [41]
RCT, postmenopausal women, aged <64, percentage of body fat: >30%, waist circumference >80 cm	EAS group (*N* = 20): stimulation at acupoint by a modulated middle-frequency interferential current electrical device vs. the control group (*N* = 21): no treatment	Body acupoints: Zusanli (ST36) and Sanyinjiao (SP6). Time and frequency: 20 minutes each time, twice a week for 12 consecutive weeks	Middle-frequency current electrical stimulation at acupoints decreased body composition (weight, waist, and hip circumference, percentage of body fat, and percentage of lean muscle mass)	Lin et al., 2010 [42]
RCT, single blind, obese females, aged 16–65, BMI >27	Auricular group (*N* = 23): received auricular acupuncture vs. the sham group (*N* = 22): received placebo needles on nonacupoint	Auricular acupoints: hunger (TG1), Shenmen (TF4), stomach (CO4), and endocrine (CO18). Time and frequency: needles kept on auricular 3 days with no pressure; two treatments per week for a total of 6 weeks with 12 treatments; ears were treated alternately	Auricular group increased ghrelin and decreased leptin levels	Hsu et al., 2009 [43]
RCT, obese female, aged 16–65, WC >90 cm, BMI >30	EA group (*N* = 22): body points vs. the sit-up exercises group (*N* = 20): sit-up exercise vs. the control group (*N* = 21): no intervention	Body acupoints: Qihai (CV6), Shuifen (CV9), Shuidao (ST28), Siman (K14), Zusanli (ST26), Fenglong (ST40), and Sanginjao (SP6). Time and frequency: EA 40 minutes each time, two treatments per week; sit-up exercises: 10 times per day, 6 weeks in total	EA group was better than the two other groups in decreasing BW, BMI, and WC	Hsu et al., 2005 [44]
Randomized crossover trial, 46 simple obese females, aged 16–65, BMI >30, WC >90 cm	A group (*N* = 24): EA + one-week washout + sit-up exercise vs. the B group (*N* = 22): sit-up exercise group + one-week washout + EA	Body acupoints: Qihai (CV6), Shuifen (CV9), Shuidao (ST28), Siman (K14), Zusanli (ST26), Fenglong (ST40), and Sanginjao (SP6). Time and frequency: EA twice a week; sit-up exercise 10 times per day, 6 weeks in total	EA was better in reducing WC and BW	Hsu et al., 2005 [45]

Acupoints embedding, laser acupuncture, or moxibustion				
RCT, double blinded, adults, aged 18–60, BMI>25, abdominal circumferences >80 cm in women and >90 cm in men	Laser acupuncture group (*N* = 19): laser acupuncture + dietary intervention vs. the sham group (*N* = 19): sham laser acupuncture + dietary intervention	Body points: Fenglong (ST40), Tianshu (ST25), Sanyinjiao (SP6), Zusanli (ST36), and Zhongwan (CV12). Time and frequency: 3 times a week for 4 weeks	Laser acupuncture had good effects on WHR, QoL scores, BMI, and appetite scores in obese patients	Sebayang et al., 2020 [46]
RCT, patient-assessor-blinded, randomized, sham-controlled crossover trial, adults aged >20, BMI >25	Laser acupuncture group (*N* = 26): laser applied by a GaAlAs semiconductor diode laser phototherapy device vs. the sham group (*N* = 26): placebo treatment under the laser with no power output; the same physician performed all laser applications	Body points: Tianshu (ST25), Zusanli (ST36), Fenglong (ST40), Neiting (ST44), Hegu (LI4), Quchi (LI11), Sanyinjiao (SP6), and Neiguan (PC6). Time and frequency: three times a week for 8 weeks; after a two-week washout period, the subjects received the treatment of the opposite group for another 8 weeks	Laser acupuncture decreased BMI, body fat percentage, WHR, waist circumference, and hip circumference compared to sham; significantly improved scores on the fullness, hunger, satiety, desire to eat, and overall well-being relative to the baseline	Tseng et al., 2016 [47]
RCT, adults, aged 25–42, BMI ≥28, WHR >0.9 in men, WHR >0.85 in women, WtHR >0.5	Polyglycolic acid sutures embedding therapy (PASET) group (*N* = 28): PASET alternatively in abdominal I group and II group vs. the control group (*N* = 23): lifestyle modification	Body acupoints: (I) Zhongwan (CV12), Shuifen (CV9), Qihai (CV6), Fuliu (CV7), Shuiguan (CV5), Mangshu (KI16), Siman (KI14), Taiyi (ST23), Tianshu (ST25), Daju (ST27), Fujie (SP14), Daheng (SP15), and Daimai (GB26); (II) Jianli (RN11), Xiawan (RN10), Guanyuan (RN4), Shiguan (KI18), Shangqu (KI17), Zhongzhu (KI15), Qixue (KI13), Huaroumen (ST24), Wailing (ST26), Shuidao (ST28), Daheng (SP15), Fuai (SP160), and Daimai (GB26). Time and frequency: the treatment cycle was repeated every 10 days, 10 weeks	PASET was better than lifestyle change; PASET significantly reduced BW, BMI, hip circumference, waist circumference, WHR, WtHR, and thickness of abdominal subcutaneous fat tissue; PASET also improved the evaluated scores in aspects of physical function, self-esteem, public distress, and sexual life, as well as decreased blood pressure, glycemia, low-density lipoprotein, uric acid, and the levels of tumor necrosis factor-alpha, interleukin-1*β*, and increased high density lipoprotein; lifestyle modification only illustrated a trend for weight decrease	Chen et al., 2019 [48]
RCT, double-blind, placebo-controlled, women, BMI ≥27, WC ≥80 cm, aged 20–65	Catgut embedding group (*N* = 45): receive acupoint catgut embedding vs. the sham group (*N* = 45): the embedding acupoints, treatment frequency, and duration were the same, but without chromic catgut strands	Body acupoints: Qihai (CV6), Shuifen (CV9), Shuidao (ST28), Siman (KI14), and Zusanli (ST36). Time and frequency: once a week, 6 weeks in total	Catgut embedding better reduced BW, WC, leptin to adiponectin ratio, and improved leptin resistance	Chen et al., 2019 [48]
RCT, overweight or obese female adults, BMI ≥25	ACET with the moxibustion group (*N* = 19): acupuncture catgut embedding + moxibustion vs. the sham group (*N* = 18): using stainless-steel needles covered with a plastic film and a cap to avoid needle insertion	Body points: Qihai (CV6), Zhongwan (CV12), Tianshu (ST25), Zusanli (ST36), Sanyinjiao (SP6), Pishu (BL20), and Shenshu (BL23). Time and frequency: catgut was implanted every 3 weeks; 5 min moxibustion was applied twice a week for a total of 6 weeks	ACET with moxibustion did not modify circulating adipokines levels; transcriptional changes in adipose tissue revealed modulation of genes participating in homeostasis control, lipid metabolism, olfactory transduction, and gamma-aminobutyric acid signaling pathway	Garcia-Vivas et al., 2016 [49]
RCT, 66 women, aged >20 with postpartum weight retention, postpartum duration <1 month, BMI >25	Laser acupuncture group (*N* = 33): using gallium aluminum arsenide laser pen vs. the sham group (*N* = 33): same acupoints without any laser output	Body acupoints: Tianshu (ST25), Shuidao (ST28), Fenglong (ST40), Daheng (SP15), Shuifen (CV9), and Sanyinjiao (SP6). Auricular points: stomach (CO4) and hunger points (TG1). Time and frequency: 12 treatment sessions; 5 times a week, approximately 3 weeks	Laser acupuncture reduced postpartum weight retention by improving BMI and BFP; no effect on WB	Hung et al., 2016 [50]
RCT, randomized, placebo-controlled, single blind, adults, aged 18–45, BMI ≥25	AM group (*N* = 22): acupuncture with moxibustion vs. the LNAM group (*N* = 10): long needle acupuncture with moxibustion vs. the EA group (*N* = 10): EA only vs. the EAM group (*N* = 20): EA with moxibustion vs. the CGM group (*N* = 25): catgut embedding with moxibustion vs. the sham group (*N* = 12): using stainless-steel needles covered with a plastic film and a cap to avoid needle insertion	Body acupoints: Qihai (CV6), Zhongwan (CV12), Tianshu (ST25), Zusanli (ST36), Sanyinjiao (SP6), Pishu (BL20), and Shenshu (BL23). Time and frequency: AM, LNAM, EA, EAM, and sham groups received two treatments per week for a total of 6 weeks (acupuncture for 20 min with or without moxibustion for 5 min in each session); for the CGM group, catgut was implanted every 3 weeks, for a total of 6 weeks, whereas moxibustion was applied twice a week	Acupoint catgut embedding therapy combined with moxibustion reduced BW, BMI, insulin, and HOMA-IR and improved insulin sensitivity	Garcia-Vivas et al., 2014 [51]

Not RCT studies presenting brain activity data				
Acute stimulation: 45 overweight or obese, 32 females and 13 males; Long-term treatment: 32 overweight or obese, 9 males and 23 females	Acute stimulation: EA (*N* = 26, 9 male) and sham (*N* = 19, 4 male) group (needles were inserted superficially to a depth of less than 5 mm and 2 cm adjacent to the selected acupoints without manipulation). Long-term treatment: EA (*N* = 17, 5 males) and sham (*N* = 15, 4 males) group (same as acute stimulation)	Body acupoints: Zhongwan (CV12), Xiawan (CV10), Tianshu (ST25), Daheng (SP15), Liangmen (ST21), Huaroumen (ST24), Wailing (ST26), Daju (ST27), and Fujie (SP14). Additional distribution acupoints: Shangjuxu (ST37), Xiajuxu (ST39), Quchi (LI11), Neiting (ST44), Zhigou (TE6), Hegu (LI4), and Fenglong (ST40). Time and frequency: acute stimulation: only once, needles in body 30 minutes, manipulation needles for 1 minute at every 15 minutes; longer time treatments: 32 minutes each time, three times a week, 6–8 weeks	Acute stimulation: both groups: weaker positive RSFC between insula and SMA/right dorsolateral-prefrontal-cortex (DLPFC) and weaker negative RSFC between insula and precuneus, stronger negative RSFC between DLPFC and DMPFC. Longer time treatments: EA group: higher BMI reduction, resting activity, and RSFC implicated in inhibitory control, gastric motility, and satiety control are associated with EA-induced weight-loss	Ren et al., 2020 [52]
19 right-handed overweight males aged 21–45, BMI 18–30	Acute acupuncture group (*N* = 10): body points vs. the sham group (*N* = 9): inserted needles superficially and immediately removed them but pretended to rotate	Body acupoints: Zusanli (ST36) and Yinlingquan (SP9). Time and frequency: rotated needles 2 minutes at a rate of 60 times per minute; needles were kept in the body during 21 minutes	Increased hypothalamus-hippocampus FC; decreased hypothalamus-thalamus FC	Von Deneen et al., 2015 [53]

ACET, acupuncture catgut embedding therapy; ASM/H2, appendicular skeletal muscle index; BMI, body mass index; BF, body fat; BFP, body fat percentage; BW, bodyweight; CCK, cholecystokinin; CRP, C-reactive protein; DLPFC, dorsolateral prefrontal cortex; DMPFC, dorsomedial prefrontal cortex; EA, electroacupuncture; FBS, fasting blood sugar; FC, functional connectivity; FFA, free fatty acids; FINS, fasting blood insulin; GLP-1, glucagon-like peptide 1; HC, hip circumference; HDL-C, high-density lipoprotein cholesterol; HFF, hepatic fat fraction; HOMA-IR, homeostatic model assessment for insulin resistance; IL-6, interleukin-6; LDL-C, low-density lipoprotein cholesterol; QoL, quality of life; RCT, randomized controlled trial; RSFC, resting-state functional connectivity; SMA, supplementary motor area; TC, total cholesterol; TFM, trunk fat mass; TG, triglycerides; TNF-*α*, tumor necrosis factor alpha; VAT, visceral adipose tissue; WC, waist circumference; WHR, waist-hip ratio; WtHR, waist-to-height ratio.

**Table 2:** Overview of rodent obesity models used in acupuncture or EA research studies.

Animal models	Obesity models	Main characteristics of the models	Animals and treatments	Acupuncture points	Main results	References
Sprague-Dawley rats	High-fat diet (HFD) during 11–15 weeks	Gut microbiota less abundant in *Bacteroides* and *Prevotella* but richer in *Bifidobacterium* and *Lactobacillus* compared to Wistar rats, Marques et al., 2015	Weaned males (3 weeks) control group (*N* = 5): standard diet group vs. the HFD group (*N* = 5): High-fat diet without treatment vs. the HFD + EA group (*N* = 5): High-fat diet + EA	Body points: Tianshu (ST25). Time and frequency: 20 minutes per time, 6 times a week, and lasted for 4 weeks.	EA inhibition of SAMs (sympathetic-associated macrophage) and the norepinephrine transporter protein SlC6a2, promoting SNS (sympathetic nervous system) activity and thermogenesis and regulating immunologic balance	Lu et al., 2020 [81]
			8-week-old male rats DIO-KOA group (*N* = 6): diet-induced obesity and knee osteoarthritis models vs. the DIO-ST36 group (*N* = 6): diet-induced obesity + EA at ST36 vs. the DIO-GB34 group (*N* = 6): diet-induced obesity + EA at GB34 vs. the DIO-ST36 + GB34 group (*N* = 6): diet-induced obesity + EA at ST36 and GB34	Body points: Liangqiu (ST34), Dubi (ST35), and Xuehai (SP10). Time and frequency: once daily for two weeks, alternately at the left and right sides of these points	All EA groups prevented obesity-induced cartilage matrix degradation and MMP expression and mitigated obesity-induced systemic and local inflammation; only ST36 + GB34 group inhibited LPS-mediated joint inflammation in obesity rats	Xie et al., 2020 [82]
			5-6-week-old male rats, 220 ± 20 g abdominal obesity HFD group (*N* = 7): nontreated vs. the acupuncture group (*N* = 7): body acupoints vs. the control group (*N* = 7): standard food	Body points: Daimai (GB26). Time and frequency: 20 minutes per session, 3 times a week, 8 weeks	Decreased BW, WC, and visceral adipose tissues, improved insulin sensitivity, glucose homeostasis, and lipid metabolism, and modified gut microbiota composition	Wang et al., 2019 [83]
			1-month-old male rats, 100–120 g, control group (*N* = 10): normal food vs. the HFD group (*N* = 10): only HFD vs. the HFD + EA group (*N* = 10): HFD + EA vs. the HFD + sham group (*N* = 10): HFD + same manner with EA but no electrical stimulation	Body points: Zusanli (ST36). Time and frequency: EA and sham 30 minutes per session, daily for 8 weeks	EA reduced BW, enhanced vagal activity, promoted acetylcholine, and activated *α*7nAChRs in white adipose tissues leading to inhibition of proinflammatory cytokine production	Jie et al., 2018 [84]
			3-4-week-old male rats, 70–90g. DIO + EA group (*n* = 18): HFD + EA vs. the DIO group (*N* = 18): HFD without treatment vs. the control group (*N* = 18): standard food	Body points: Tianshu (ST25), Zhongwan (CV12), Sanyinjiao (SP6), and Zusanli (ST36). Time and frequency: 30 min per day during 30 days, with a 2-day break after every 5-day treatment	EA reduced BW, body fat and body fat rate, induced hypothalamic Tsc1 promoter demethylation, and inhibited the mTORC1 signaling pathway	Leng et al., 2018 [85]
			4-week-old male rats, 50–70 g. EA group (*N* = 10): HFD + EA vs. the DIO group (*N* = 10): HFD without treatment	Body points: Zusanli (ST36) and Neiting (ST44). Time and frequency: 15 minutes per session, 3 times a week, 4 weeks in total	EA reduced body, liver, and fat pad weight, decreased hepatic TG and total cholesterol, fatty droplet accumulation, and serum concentrations of ALT and AST, restored phosphorylation levels of AMPK (Thr172) and ACC (Ser79) that appear to be mediated through AMPK signaling pathways	Gong et al., 2016 [86]
			Male rats, 45–55 g, low-fat diet group (*N* = 10): fed only low-fat food vs. the HFD group (*N* = 10): high-fat diet vs. the EA group (*N* = 10): high fat food + EA vs. the OLST group (*N* = 10): high-fat food + orlistat	Body points: Zusanli (ST36) and Quchi (LI11). Time and frequency: 20 minutes per session, once a day for 28 days	EA reduced BW, homeostasis model assessment-insulin resistance index, adipocyte diameters, and neuroprotein Y/agouti-related protein and protein tyrosine phosphatase 1B levels; the OLST group had watery stools and yellow hairs	Liu et al., 2016 [87]
			54-week-old male rats, 80–100 g, high-fat food for 4 weeks; EA group (*N* = 12): body acupoints vs. the DIO group (*N* = 12): restricted restrained for 20 min without EA stimulation vs. the chow fed group (*N* = 13): normal rat fed standard food	Body points: Tianshu (ST25) and Zusanli (ST36). Time and frequency: 20 minutes once a day, 6 days per week, four weeks in total	EA tended to reduce weight growth and regulated hypothalamic LKB1-AMPK-ACC signaling	Xu et al., 2015 [88]
			CEA group (*N* = 10): electrical stimulation at acupoints by surgically implanted electrodes with continuous pulses vs. the sham group (*N* = 10): no electric stimulation	Body points: Zusanli (ST36). Time and frequency: 2 hours daily for 2 months	CEA reduced BW, food intake, vagal activity, epididymal fat pad weight, postprandial blood glucose, and HbA1c; increased sympathetic activity, fasting plasma level of glucagon-like peptide-1 (GLP-1) and peptide YY	Liu et al., 2015 [89]
			4-week-old male rats, 130 ± 12 g, control group (*N* = 5): standard food vs. the DIO group (*N* = 5): high-fat diet: no treatment vs. 3-time EA group (*N* = 5): 3 times EA treatment vs. the 7-time EA group (*N* = 5): 7 times EA treatment	Body points: Zusanli (ST36). Time and frequency: 20 min per session, daily	Seven times a week, EA reduced the tumor necrosis factor-*α* (TNF-*α*), IL-6, monocyte chemotactic protein-1 (MCP-1), and CD68 mRNA expression and improved adipose tissue inflammatory via attenuation of lipogenesis signaling	Chorng-Kai et al., 2014 [90]
			1-month-old newly weaned male rats, 50–70 g, normal group (*N* = 12): standard food vs. the unoperated obese group (*N* = 12): high-fat diet without treatment vs. the implantation group (*N* = 12): high-fat diet + catgut implantation	Body points: Zusanli (ST36) and Neiting (ST4). Time and frequency: once a week for 4 weeks	Catgut implantation at acupoint decreased BW, serum leptin, and INS levels and increased hypothalamic leptin and INS levels and OB-R gene expression	Yan et al., 2012 [91]
			1-month-old male rats, 50–70 g, control group (*N* = 20): standard diet vs. the obese group (*N* = 10): high-fat diet vs. the obese EA group (*N* = 10): high-fat diet obese rats + EA	Body points: Zusanli (ST36) and Neiting (ST44). Time and frequency: 30 minutes per session, 3 times a week for 4 weeks	EA reduced BW, decreased plasma leptin levels, and increased leptin receptor expression in the hypothalamus	Gong et al., 2012 [92]
			21-day-old male rats, DIO group (*N* = 17): diet-induced obesity vs. the restraint group (*N* = 10): high-fat diet + restraint 30 minutes without EA vs. the sham group (*N* = 10): nonacupoint located on the proximal part of the tail vs. the EA group (*N* = 17): high-fat diet obese rats + EA	Body points: Zusanli (ST36) and Sanyinjiao (SP6). Time and frequency: 30 minutes per session, 7 times a week for 2 weeks	EA group decreased food intake and BW, increased peptide levels of *α*-MSH and mRNA levels of its precursor POMC in the ARH neurons, and elevated the CSF content of *α*-MSH	Guan et al., 2003 [93]
			14 weeks chronic high-fat diet, male rats, 45–55 g, control group (*N* = 20): standard food vs. 2 Hz EA group (*N* = 10): HFD + EA with 2 Hz current vs. 100 Hz EA group (*N* = 10): HFD + EA with 100 Hz current vs. the DIO group (*N* = 10): HFD without treatment	Body points: Zusanli (ST36) and Sanyinjiao (SP6). Time and frequency: 3 times a week for 4 weeks	EA reduced BW and energy intake and increased the expression of CART in ARC	Tian et al., 2005 [94]

Wistar rats	High-calorie diet during 7–16 weeks	Increased energy ingestion, weight gain, body fat mass, mesenteric adipocyte's size, adiponectin, and leptin plasma levels and decreased oral glucose tolerance more evident or detected earlier than in SD rats, Marques et al., 2015	Male Wistar rats (180–220 g), HFD group (*N* = 7): high-fat diet vs. the GOH group (*N* = 7): HFD + garlic oil (GO) Shenque 100 mg/kg/day vs. the GOM group (*N* = 7): HFD + GO Shenque 50 mg/kg/day vs. the GOL group (*N* = 7): HFD + GO Shenque 25 mg/kg/day vs. the GOO group (*N* = 7): HFD + GO oral administration 50 mg/kg/day vs. the naive group (*N* = 7): fed with normal diet	Body points: garlic oil injection on Shenque (CV8). Time and frequency: once every day totally for 6 weeks	Acupoint garlic oil injection was better than oral administration in decreasing BW, fat mass, cholesterol, triglyceride low density, and lipoprotein concentrations	Zhang et al., 2019 [95]
			8-week-old male rats, 200 ± 20 g, normal group (*N* = 10): normal diet vs. obesity group (*N* = 10): high-calorie diet vs. the EA group (*N* = 10): body points vs. the resveratrol group (*N* = 10): 95% resveratrol 200 mg/kg	Body points: Zusanli (ST36), Fenglong (ST40), Zhongji (CV3), Juliao (ST3), and Guanyuan (CV4). Time and frequency: 10 minutes per session, 3 times a week for 8 weeks	EA activated Sirt1 and Sirt1-dependent deacetylation of histone (H3K9); activation of Sirt1 could lead to deacetylation of NF-*κ*B; EA regulated hyperlipidemia and insulin resistance (IR)	Luo et al., 2018 [96]
			80 male Wistar rats, average weight 250 ± 10 g, SDH group (*N* = 10): standard diet/manipulation vs. the SDNO group (*N* = 10): standard diet nonacupoint vs. the SDP36 group (*N* = 10): standard diet/acupoint ST36 vs. the SDP25 group (*N* = 10): standard diet/acupoint ST25 vs. the HDH group (*N* = 10): hypercaloric diet/manipulation vs. the HDNP group (*N* = 10): hypercaloric diet/nonacupoint vs. the HDP36 group (*N* = 10): hypercaloric diet/acupoint ST36 vs. the HDP25 group (*N* = 10): hypercaloric diet/acupoint ST25	Body points: Zusanli (ST36) and Tianshu (ST25); pharmacopuncture with the application of bee venom (Sigma-Aldrich®, 0.025 mg/kg) on points time and frequency: injection once a day for 12 weeks	Pharmacopuncture groups had reduced bodyweight, perirenal fat weight and HDP36, lower abdominal fat weight, reduced cholesterol and glucose levels, as well as regulated behavioral parameters	Pontes et al., 2015 [97]
	VMH (hypothalamic) lesions made by electrocoagulation in stereotaxic coordinates under anesthesia		Male Wistar SPF/VAF rats, 4-week-old (80–90 g) and 7-8-week-old (150–180 g); increased bodyweight 2 weeks after the intervention obesity group (*N* = 41): (both hypothalamic 20 + dietary 21) obese rats + auricular stimulation vs. the control group (*N* = 21): mature normal rats + auricular stimulation auricular stimulation: stimulating electrode was a stainless steel ear needle	Auricular points: on the ipsilateral vagal innervated region of the auricle; equivalent to the cavum concha in human: heart (CO15), lung (CO14), spleen (CO13), and trachea (CO16)	Auricular acupuncture stimulation modulated feeding-related hypothalamic neuronal activity of experimental (both hypothalamic and dietary) obese rats, in correlation with the degree of obesity; auricular acupuncture stimulation may not reduce appetite, but is more likely to modulate satiation	Shiraishi et al., 1995 [98]
	Just different ages without intervention		Nonobese: 290 g or less, obese: 450 g or over auricular acupuncture group (*N* = 10): 5 nonobesity + 5 obesity + acupuncture vs. the control group (*N* = 10): 5 nonobesity + 5 obesity, same as the acupuncture group but with no needles vs. the sham group (*N* = 5): obese rats, needles were inserted into nonacupuncture points; auricular acupuncture stimulation: 3 mm long needles inserted into acupoints and fastened in place with (7–0) nylon thread sutures	Auricular points: pylorus (CO3), lung (CO14), trachea (CO16), stomach (CO4), esophagus (CO2), endocrine (CO18), and heart (CO15). Time and frequency: needles were inserted into the ear for 21 days	Auricular acupuncture induced evoked potentials in the hypothalamic ventromedial nucleus (HVM), the satiety center, and reduced BW	Asamoto et al., 1992 [99]

C57BL/6 mice	High-fat diet, 8–12 weeks	One of the oldest inbred mouse strains; careful because some substrains, such as C57BL/6JRj mice, are protected against DIO, Kern et al., 2012	7-week-old mice, 19–22 g, control group (*N* = 7): normal diet vs. the obesity group (*N* = 7): HFD vs. the acupoint catgut embedding group (*N* = 7): HFD + embedding vs. the sham group (*N* = 7): HFD + using similar equipment to insert the empty needle at same acupoint without catgut implantation; normal TRPV1 KO group (*N* = 7): TRPV1 knockout mice + normal diet vs. the obesity TRPV1 KO group (*N* = 7): TRPV1 knockout mice + HFD	Body points: Zusanli (ST36). Time and frequency: acupoint catgut embedding and the sham group both received once treatment per week, 8 weeks in total	Acupoint catgut embedding decreased BW, increased MAPK pathway-related proteins (glucose, leptin and insulin plasma levels, and protein molecule density of TRPV1)	Inprasit et al., 2020 [100]
			3-week-old male C57BL/6J mice. HFD group (*N* = 9): HFD vs. the HFD + EA group (*N* = 14): high-fat food + EA vs. the normal group (*N* = 8): standard food vs. normal + EA group (*N* = 13): standard food + EA	Body points: Zusanli (ST36) and Neiting (ST44). Time and frequency: 30 min once a day, 6 days a week, 4 weeks in total	EA reduced food intake, BW, TG, and cholesterol and improved glucose tolerance in fat mice, activated sympathetic nerves via p-TH, A2AR, and *β*3AR in white adipose tissue	Lu et al., 2019 [101]
			11-week-old mice, 20.4 ± 0.52 g, normal group (*N* = 6): standard food vs. the model group (*N* = 6): obese mice without intervention vs. the A7 group (*N* = 6): obese mice + 7 d EA vs. the A14 group (*N* = 6): obese mice + 14d EA vs. the A21 group (*N* = 6): obese mice + 21 d EA vs. the A28 group (*N* = 6): obese mice + 28 d EA	Body points: Tianshu (ST25), Guanyuan (CV4), Zusanli (ST36), and Sanyinjiao (SP6). Time and frequency: 10 minutes per session, daily	EA regulated the structures and functions of the intestinal microbiota and altered the bacterial diversity and metabolic genes to establish a new balance	Si et al., 2018 [102]
			4-week-old C57BL/6 J mice normal group (*N* = 20): standard diet vs. the EA group (*N* = 28): high-fat diet + EA vs. the control group (*N* = 28): HFD without treatment	Body points: Zusanli (ST36) and Neiting (ST44). Time and frequency: 30 minutes per session, once a day, six days a week, 4 weeks in total	EA decreased WAT/bodyweight ratio, increased number of UCP1-immunoreactive paucilocular adipocytes, increased expressions of brown adipose tissue (BAT) markers, including UCP1, COX4il, and Nrtf1 in the WAT, and decreased acetylation of PPAR*γ*	Shen et al., 2014 [103]

OLETF rats (Otsuka Long-Evans Tokushima Fatty)	Genetic obesity	Spontaneous mutation, lacking expression of functional CCK1; early onset overeating-induced obesity, An et al., 2007. mRNA expressions of several insulin signaling-related molecules IRS1, IRS2, Akt2, aPKC*ζ*, and GLUT4 were decreased in OLETF rats compared to SD controls, Huang et al., 2016	OLETF rats, male, 360.0 ± 35.6 g, genetic models of hyperglycemia at 5 weeks of age; SD rats, 10-week-old male, 222.6 ± 22.6 g, control group (*N* = 8): SD rats vs. the hyperglycemic model group (*N* = 8): OLETF rats vs. the acupuncture + hyperglycemic model group (*N* = 8): OLETF rats + acupuncture	Body points: Neiguan (PC6), Zusanli (ST36), Sanyinjiao (SP6), and Shenshu (BL23). Time and frequency: 20 min, once a day, 21 days in total	Acupuncture decreased FPG, FINS, C-peptide, and HOMA-IR and modulated insulin signaling molecules (IRS1, IRS2, Akt2, aPKC, and GLUT4)	Huang et al., 2016 [104]
			Male OLETF 5-week-old rats, AcOLETF group (*N* = 12): OLETF + acupuncture vs. the OLETF group (*N* = 18): no treatment vs. the control LETO group (*N* = 10): Long-Evans Tokushima Otsuka without treatment	Body points: Zhongwan (CV12), Tianshu (ST25), Qihai (CV6), Ganshu (BL18), Pishu (BL20), and Shenshu (BL23). Time and frequency: acupuncture stimulation was applied twice a week	Acupuncture reduced blood glucose levels, but not bodyweight	Nakamura et al., 2014 [105]

Long-Evans rats	11-week high-fat diet	Appropriate obesity model but discrepancies in gene expression alterations between DIO Long-Evans rats and obese humans, particularly in the metabolic pathways, Li et al., 2008	Male Long-Evans rats at 3 weeks as obese rats, 11 weeks standard feed as lean rats, EA group 1 (5 lean + 7 obese): received EA, EA group 3 (5 lean + 7 obese): received EA vs. the group 2 (5 lean + 7 obese): no treatment, group 4 (5 lean + 7 obese): no treatment	Body points: Zhongwan (CV12) and Guanyuan (CV4). Time and frequency: 30 minutes per session, 3 times a week, 2 weeks	EA group 1 had significantly higher serum interleukin-10 and tumor necrosis factor-*α* than group 2, while EA group 3 serum leptin was higher than that of group 4; white adipose tissue interleukin-10 and adiponectin: leptin ratio was higher for EA group 1 than group 2	Liaw and Peplow, 2016 [106]

Zucker fatty rats	Genetic obesity	Mutated leptin receptor genes, two homozygous fa/fa “fatty” alleles resulting in leptin insensitivity, Boomhower et al., 2013, and Castonguay et al., 1982	12–14-week-old male Zucker fa/fa rats, 3-week-old male Long-Evans rats, Zucker EA group (*N* = 5): obese rats treated with EA vs. the control obesity Zucker group (*N* = 7): obese rats without treatment vs. the Long-Evans EA group (*N* = 7): obese Long-Evans rats treated with EA vs. the control obesity Long-Evans group (*N* = 7): obese Long-Evans rats without treatment	Body points: Zhongwan (CV12) and Guanyuan (CV4). Time and frequency: alternate weekdays (Monday, Wednesday, and Friday) over 2 weeks, 6 sessions	EA altered the balance of pro/anti-inflammatory adipokines in two obesity rat models and indicated the possibility for other alterations at the levels of adipokines by EA. In Zucker rats, there was an increase in the IL-10: TNF-*α* ratio. In obese Long-Evans rats, the adiponectin:leptin ratio was decreased by EA	Liaw and Peplow, 2016 [107]
			Male obese Zucker fatty rats, 12–14 weeks age, EA group (*N* = 5): obesity Zucker rats treated with EA vs. the control group (*N* = 7): obesity Zucker rats without treatment	Body points: Zhongwan (CV12) and Guanyuan (CV4). Time and frequency: alternately weekdays, six applications, over 2 weeks	EA decreased serum TNF-*α* but produced no significant alterations in serum leptin, adiponectin, or IL-10 and no significant effect on the levels of these four adipokines in white adipose tissue. Compared with the control animals, no significant change in BW	Liaw and Peplow, 2016 [108]

Stat5NKO mice: (Stat5-deleted mice)	Genetic engineering	Deletion of Stat5 from neurons	12-week-old Stat5NKO and Stat5fl/fl mice with bodyweight 20% heavier than that of Stat5fl/fl mice, Stat5NKO group (*N* = 10): restrained for 30 min without EA vs. the Stat5NKO + EA group (*N* = 10): EA treatment vs. the Stat5fl/fl group (*N* = 10): restrained for 30 min without EA vs. the Stat5fl/fl + EA group (*N* = 10): EA treatment	Body points: Zusanli (ST36) and Neiting (ST44), both sides were used alternatively; time and frequency: once a day, 6 times a week, total 4 weeks	Reduced bodyweight and ratio of epididymal white adipose tissue (epi-WAT) to bodyweight, increased cold endurance, decreased glucose plasma levels, triglyceride, and cholesterol, and reversed altered gene expressions in the hypothalamus and epi-WAT	Fu et al., 2017 [109]

Ob/ob mice (obese leptin-deficient)	Genetic engineering	Obese leptin-deficiency	C57BL/6J male mice, 4-week-old male (ob/ob) mice, control group (*N* = 5): C57BL/6J mice vs. the 3 ob/ob mice + EA group (*N* = 5): 3 consecutive EA at 22 weeks vs. the 7 ob/ob mice + EA group (*N* = 5): 7 consecutive EA from 21 weeks to 22 weeks	Body points: Zusanli (ST36). Time and frequency: 10 minutes one time, 3 times a week, once every second day	EA prevented weight gain through HIF-1*α*- (hypoxia-inducible factors-1*α*-) dependent pathways and inflammatory response in obese adipose tissues and reduced hypoxia-related inflammation-related genes	Wen and Lee, 2015 [110]

Obesity-prone (OP) rats	Special genetic phenotype	OP rats were hyperphagic and showed a 20% weight gain over OR rats at 15 weeks with standard diet, associated with a 50% reduction in basal extracellular dopamine; dopamine impairment was apparent at birth, Geiger et al., 2008	OP rats (7-week-old, 180–200 g), obesity-resistant (OR) rats; age-matched control strain, control group (*N* = 10): OP and OR rats + no treatment vs. the EA group (*N* = 10): OP and OR rats + EA vs. the sham group (*N* = 10): OP and OR rats + sham EA (needles were taped on the surface of acupoint ST36 without electrical stimulation)	Body points: Zusanli (ST36). Time and frequency: 20 minutes daily, 7 days in total	EA reduced food intake and BW, produced an upregulation of anorexigenic factor POMC production in NTS/HN; EA-induced expression of TRPV1-nNOS in ST36 and NTS/gracile nucleus is involved in the signal transduction of EA stimuli via somatosensory afferents-medulla pathways	Ji et al., 2013 [111]

TRPV1−/− mice	Genetic engineering	TRPV1 KO mice exacerbate more obesity and insulin resistance associated with HFD and aging than wild-type mice, Lee et al., 2015	8–12-week-old adult male C57/B6 mice, TRPV1−/− mice control group (*N* = 7): C57/B6 mice without treatment vs. the EA group (*N* = 7): C57/B6 mice + EA vs. the sham-EA group (*N* = 7): C57/B6 mice + similar protocol of EA was applied to gluteal muscle nonacupoint vs. the TRPV1−/− group (*N* = 7): TRPV1−/− mice without treatment vs. the TRPV1−/− mice + EA group (*N* = 7): TRPV1−/− mice + EA	Body points: Zusanli (ST36). Time and frequency: 15 min per session, 4 weeks in total	EA decreased BW and visceral WAT weight and increased protein levels of TRPV1, pPKA, pPKC, and pERK in the dorsal root ganglion and spinal cord	Choowanthanapakorn et al., 2015 [112]

ALT, alanine aminotransferase; ARC, arcuate nucleus; ARH, arcuate nucleus of the hypothalamus; AST, aspartate aminotransferase; CART, cocaine and amphetamine-regulated transcript peptide; CSF, cerebral spinal fluid; DIO, diet-induced obesity; EA, electroacupuncture; FPG, fasting plasma glucose; HFD, high-fat diet; HOMA-IR, homeostasis model assessment-estimated insulin resistance; HN, hypoglossal nucleus; HRV, heart rate variability; INS, insulin; IR, insulin resistance; KO, knock out; NTS, nucleus tractus solitarius; OLETF, Otsuka Long-Evans Tokushima Fatty; OP, obesity-prone; OR, obesity-resistant; POMC, proopiomelanocortin; qRT-PCR, quantitative RT-PCR; RNA-seq, ribonucleic acid sequencing; RT-PCR, reverse transcription polymerase chain reaction; SD, standard diet; TG, hepatic triglyceride; TRPV1, transient receptor potential cation channel subfamily V member 1; VMH, ventromedial hypothalamic area; WAT, white adipose tissue; WB, Western blot.
